# Vascular derived endothelin receptor A controls endothelin-induced retinal ganglion cell death

**DOI:** 10.1038/s41420-022-00985-8

**Published:** 2022-04-16

**Authors:** Olivia J. Marola, Gareth R. Howell, Richard T. Libby

**Affiliations:** 1 Department of Ophthalmology, Flaum Eye Institute, University of Rochester Medical Center, Rochester, NY USA; 2 Cell Biology of Disease Graduate Program, University of Rochester Medical Center, Rochester, NY USA; 3 The Center for Visual Sciences, University of Rochester, Rochester, NY USA; 4 The Jackson Laboratory, 600 Main Street, Bar Harbor, ME USA; 5 Department of Biomedical Genetics, University of Rochester Medical Center, Rochester, NY USA

**Keywords:** Cell death in the nervous system, Apoptosis

## Abstract

Endothelin (EDN, also known as ET) signaling has been suggested to be an important mediator of retinal ganglion cell (RGC) death in glaucoma. Antagonism of EDN receptors (EDNRA and EDNRB, also known as ET-A and ET-B) prevented RGC death in mouse models of chronic ocular hypertension, and intravitreal injection of EDN ligand was sufficient to drive RGC death. However, it remains unclear which cell types EDN ligands directly affect to elicit RGC death. Multiple cell types in the retina and optic nerve express EDNRA and EDNRB and thus could respond to EDN ligands in the context of glaucoma. Here, we systematically deleted *Edn* receptors from specific cell types to identify the critical EDN receptor mediating RGC death in vivo. Deletion of both *Ednra* and *Ednrb* from retinal neurons (including RGCs) and macroglia did not prevent RGC loss after exposure to EDN1 ligands, suggesting EDN1 ligands cause RGC death via an indirect mechanism involving a secondary cell type. Deletion of *Ednra* from the full body, and then specifically from vascular mural cells, prevented EDN1-induced vasoconstriction and RGC death. Together, these data suggest EDN ligands cause RGC death via a mechanism initiated by vascular mural cells. It is possible RGC death is a consequence of vascular mural cell-induced vasoconstriction and its pathological sequelae. These results highlight the potential importance of neurovascular dysfunction in glaucoma.

## Introduction

Glaucoma is a neurodegenerative condition affecting the output neurons of the retina—the retinal ganglion cells (RGCs). One of the most important risk factors for developing glaucomatous neurodegeneration is elevated intraocular pressure (IOP) [1]. To date, elevated IOP is the only clinically treatable component of glaucoma, and unfortunately, normalizing IOP does not prevent glaucoma progression or development in many patients [2]. Therefore, understanding the molecular signaling pathways that lead from ocular hypertensive injury to RGC death is critical for understanding the pathobiology of glaucoma. Recent evidence has suggested the importance of RGC-extrinsic signaling events (e.g., neuroinflammation, neurovascular dysfunction) in triggering glaucomatous RGC injury [3–6]. Molecular clustering analysis of ocular hypertensive DBA/2J retinas and optic nerves revealed several candidate mechanisms that are potentially critical in driving RGC injury in glaucoma, including activation of the endothelin system [5, 7].

The endothelin system is a family of three ligands (EDN1, 2, and 3, also known as ET-1, 2, and 3) and two G-protein coupled receptors (EDNRA and EDNRB, also known as ET-A and ET-B). The canonical role of the endothelin system is to regulate blood flow and vasoconstriction. Potent vasoconstriction occurs when EDN ligands bind to EDNRA [8–11], which is highly expressed by vascular mural cells [6, 12, 13], including smooth muscle cells [14–17] and pericytes [18, 19]. EDNRB is expressed by vascular endothelial cells [20–22], and is thought to mediate vasorelaxation in response to ligand binding. Many organ systems, including the central nervous system, use the endothelin system to maintain normal physiology [23, 24].

As with many signaling systems that have a physiological role, endothelin signaling has also been broadly implicated in the pathophysiology of numerous diseases, including retinal diseases and glaucoma [25, 26]. EDN ligands and receptors are known to be expressed by glaucoma-relevant cell types. EDN ligands have been shown to be expressed by retinal and optic nerve macroglia [6, 27] and myeloid-derived cells [5, 6], while both EDN receptors are expressed by retinal neurons (including RGCs) [12, 14, 25, 28, 29] and macroglia [26, 30–33]. Endothelin signaling has been hypothesized to play a role in human glaucoma. Levels of EDN ligand were found to be higher in the aqueous humor and plasma of glaucoma patients [34, 35]. Changes in blood flow have been documented in human [36–39] and animal models [3, 5] of glaucoma, and it is hypothesized that these changes could be important factors in the development and progression of glaucoma. Animal models of ocular hypertension have also indicated a potential role for endothelin signaling in glaucoma. *Edn* ligands and receptors were significantly upregulated in retinas and optic nerve heads of ocular hypertensive DBA/2J mice before the onset of glaucomatous neurodegeneration [3, 5, 6]. Similar patterns of endothelin system upregulation were found in models of acutely induced ocular hypertension [28] and after glaucoma-relevant optic nerve crush [40]. EDN ligands are sufficient to cause RGC death— intravitreal injection or transgenic overexpression of EDN ligands caused significant RGC loss and axonal degeneration [3, 5, 11, 12, 41–44]. Caspase 3 activation in RGCs and later RGC loss after EDN1 exposure was dependent upon JUN activation, similar to RGC death after glaucoma-relevant injuries including optic nerve crush [45] and ocular hypertension [46]. Importantly, pan-antagonism of EDN receptors with Bosentan or Macitentan conferred significant protection from glaucomatous neurodegeneration in DBA/2J ocular hypertensive mice [5, 6]. Thus, targeting endothelin signaling may have potential as a neuroprotective treatment for glaucoma.

Despite their apparent role in glaucoma pathology, it is unclear how EDN ligands act in the retina or optic nerve to ultimately drive RGC death. It is possible EDN ligands cause RGC death directly via RGC-expressed EDN receptors, as has been demonstrated in vitro [12]. But it is also possible EDN ligands bind to receptors expressed by astrocytes or vasculature, thereby triggering a neurotoxic response. Understanding the cell types important in EDN-induced RGC death will provide insight into early, critical pathways of glaucomatous neurodegeneration and can identify potential therapeutic targets for neuroprotective glaucoma treatments. The present work utilized cell-specific deletions of *Ednra* and/or *Ednrb* to investigate the mechanisms by which EDN ligands drive RGC death in vivo.

## Results

### EDN ligand did not act through RGC- or macroglia-expressed EDN receptors to cause RGC death

Intravitreal delivery of EDN ligand was sufficient to drive RGC death [3, 5, 11, 12, 41, 42]. Studies have suggested EDN ligands cause RGC death directly. Primary RGCs in culture underwent cell death after EDN ligand exposure [12, 25], suggesting EDN ligands can bind to RGC-expressed EDN receptors and drive cell death. There is evidence to suggest RGCs express both EDNRB [6, 12, 13, 28, 29] and EDNRA [12, 28, 29], therefore, EDN ligands could bind to either or both RGC-expressed EDN receptors to directly cause RGC death. To investigate whether EDN ligands affect RGCs directly, Six3-cre was used to recombine homozygous floxed alleles of *Ednra* and/or *Ednrb*. Six3-cre is well known to recombine floxed alleles in retinal neurons, including in 80% of RGCs [45]. Of note, studies have reported Six3-cre-mediated recombination of floxed alleles in macroglia (astrocytes and Müller glia), but not in vascular cells [47–49]. Macroglia (Müller glia and astrocytes) are known to robustly express EDNRB [6], and some studies have shown EDNRA expression by astrocytes [31–33]. Therefore, EDN ligand could feasibly bind to EDN receptors expressed by macroglia to ultimately cause RGC death.

**Fig. 1:**
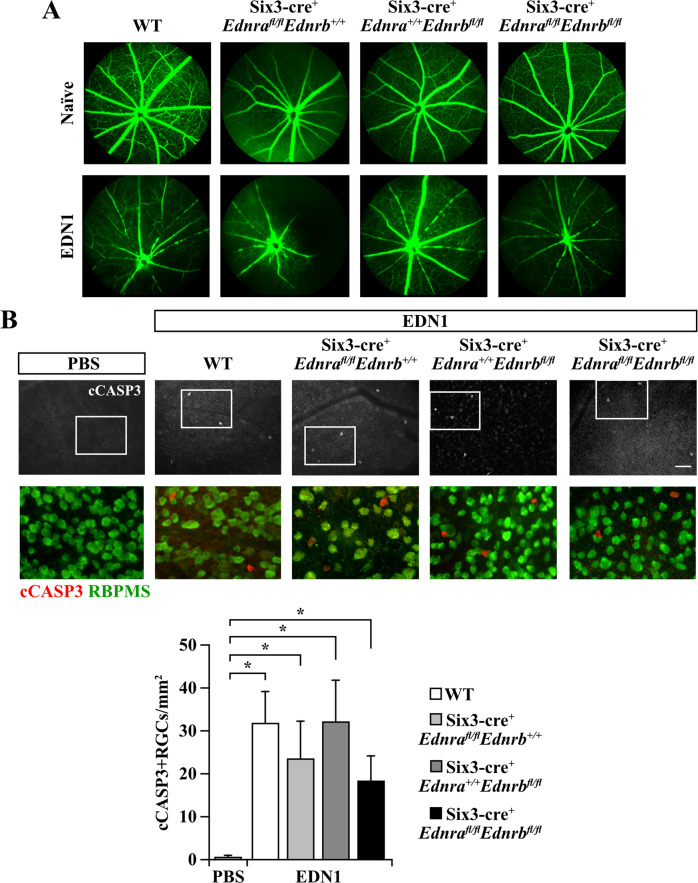
EDN1 did not cause RGC death directly or via macroglia-expressed receptors. **A** Fluorescein angiography of retinal vasculature in naive and EDN1-injected eyes from WT, Six3-cre^+^*Ednra*^*fl/fl*^*Ednrb*^*+/+*^, Six3-cre^+^*Ednra*^*+/+*^*Ednrb*^*fl/fl*^, and Six3-cre^+^*Ednra*^*fl/fl*^*Ednrb*^*fl/fl*^ animals. Deletion of either or both *Edn* receptors with Six3-cre did not prevent EDN1-induced vasoconstriction (*n* ≥ 3). **B** Retinal flat mounts and quantification of cleaved caspase 3+ (cCASP3+, red) RBPMS+ (green) cells from WT, Six3-cre^+^*Ednra*^*+/+*^*Ednrb*^*fl/fl*^, Six3-cre^+^*Ednra*^*fl/fl*^*Ednrb*^*+/+*^, Six3-cre^+^*Ednra*^*fl/fl*^*Ednrb*^*fl/fl*^ 5 days post-EDN1 or PBS (vehicle control) injection. Each genotype group had significant increases in cCASP3+ RGCs compared to PBS controls. No significant difference in cCASP3+ RGCs was observed between genotype groups after EDN1. cCASP3+ RGCs/mm^2^ ± SEM: PBS: 0.8 ± 0.3, WT: 32.0 ± 7.2, Six3-cre^+^*Ednra*^*fl/fl*^*Ednrb*^*+/+*^: 23.7 ± 8.6, Six3-cre^+^*Ednra*^*+/+*^*Ednrb*^*fl/fl*^: 32.3 ± 9.5, Six3-cre^+^*Ednra*^*fl/fl*^*Ednrb*^*fl/fl*^: 19.8 ± 6.0 (*n* ≥ 7 per genotype, *P < 0.05, Kruskal–Wallis test). Scale bars, 50 μm.

To determine whether neuronal and/or macroglial EDN receptors are required for EDN-induced RGC death, EDN1 was intravitreally injected into the eyes of WT (Six3-cre^−^*Ednra*^+/+^*Ednrb*^+/+^, Six3-cre^+^*Ednra*^*fl/fl*^*Ednrb*^*+/+*^, and Six3-cre^+^*Ednra*^*+/+*^*Ednrb*^*fl/fl*^ mice. PBS was injected into the contralateral eye as a vehicle-matched control. As expected,` Six3-cre-mediated deletion of *Edn* receptors did not interfere with EDN1-induced vasoconstriction (vascular smooth muscle-expressed EDNRA is known to induce vasoconstriction upon ligand binding [8–11]). Intravitreal EDN1 injection caused similar levels of vasoconstriction in WT, Six3-cre^+^*Ednra*^*fl/fl*^, Six3-cre^+^*Ednrb*^*fl/fl*^, and Six3-cre^+^*Ednra*^*fl/fl*^*Ednrb*^*fl/fl*^ retinas (Fig. 1A). Previous reports have shown EDN1 caused caspase 3 activation (cleavage; cCASP3) in RGCs 5 days post-intravitreal injection, which corresponded to RGC dropout by 28 days [44]. Genetic manipulations that prevented later RGC dropout also prevented early caspase 3 activation [44]—a pattern which is also observed after other glaucoma-relevant injuries [46, 50]. Therefore, the presence of cCASP3+ RGCs was used to assess RGC injury after EDN1 injection. As reported previously, intravitreal injection of PBS did not drive appreciable caspase 3 activation in RGCs, and intravitreal injection of EDN1 ligand drove significant caspase 3 activation in RGCs (Fig. 1B). Surprisingly, deletion of either *Ednra* or *Ednrb* from retinal neurons (including RGCs) and macroglia did not prevent EDN1-induced caspase 3 activation (cleavage) in RGCs (Fig. 1B). To address the possibility that both EDN receptors expressed by retinal neurons and/or macroglia are required to cause RGC death, EDN1 was injected into the eyes of Six3-cre^+^*Ednra*^*fl/fl*^*Ednrb*^*fl/fl*^ mice. Deletion of both *Edn* receptors from macroglia and retinal neurons did not prevent caspase 3 activation in RGCs in response to EDN1 (Fig. 1B). These data suggest EDN1 ligands did not directly affect neurons (including RGCs) or macroglia to drive RGC death. Rather, EDN1 ligands acted through either EDNRA or EDNRB expressed by a different cell type. These results necessitated the identification of the EDN receptor, regardless of the cell type expressing it, which ultimately drives EDN1-induced RGC death.

### Endothelin ligand acted through non-neuronal, non-macroglial EDNRA to elicit RGC death

Given EDN1 did not elicit RGC death via RGC- or macroglia-expressed EDN receptors, EDN1 must directly affect a different cell type through either EDNRA or EDNRB. Beyond retinal neurons and macroglia, *Ednra* is known to be expressed by vascular mural cells [6, 12–19], and *Ednrb* is known to be expressed by endothelial cells [20–22]. It is also possible *Ednra* and/or *Ednrb* are expressed at low levels by another cell type (e.g., myeloid cells) and are able to pathologically respond to EDN ligand exposure. To determine whether EDN acts through EDNRB or EDNRA to cause RGC death, EDN1-induced RGC death was assessed in mice with global deletions of *Ednra* or *Ednrb* (Cag-creER^T2+^*Ednra*^*fl/fl*^ and Cag-creER^T2+^*Ednrb*^*fl/fl*^ mice were treated with tamoxifen to produce full-body knockouts).

**Fig. 2:**
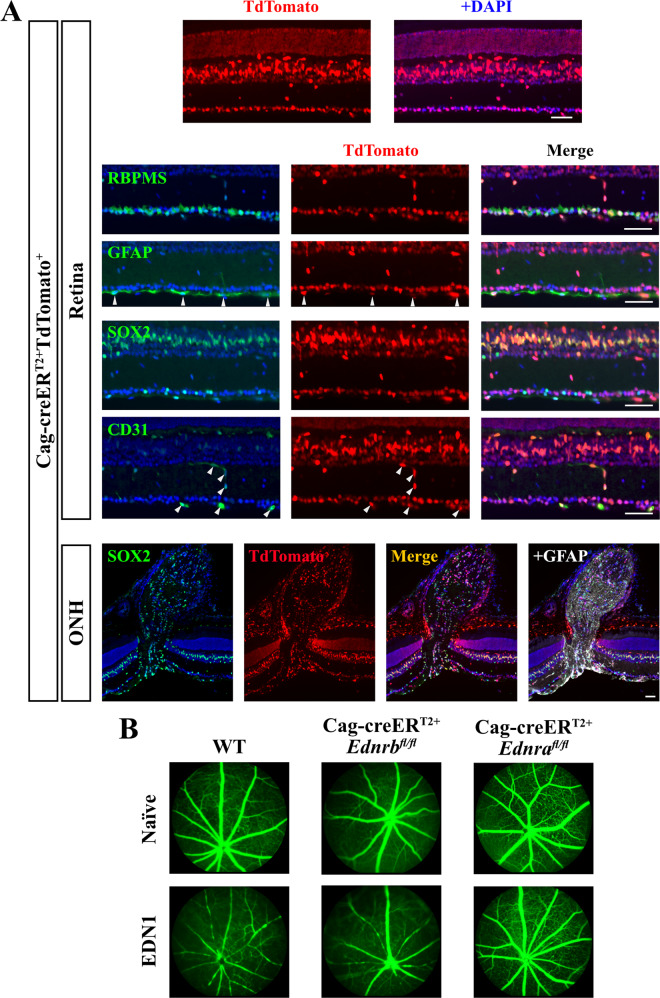
Cag-creER^T2^ robustly recombined floxed alleles in retinal and optic nerve DAPI+ cells. **A** Cag-creER^T2+^Tdtomato^+^ retinal and optic nerve head sections depicting cell-type-specific expression of Tdtomato. Cag-creER^T2^ robustly recombined floxed alleles in DAPI+ cells, including RBPMS+ RGCs, SOX2+ Müller glia, GFAP+ retinal astrocytes, CD31+ vascular cells, and SOX2+ GFAP+ optic nerve head (ONH) astrocytes (*n* = 3). Scale bars, 50 μm. **B** Fluorescein angiography of retinal vasculature in naive and EDN1-injected eyes from WT, Cag-creER^T2+^*Ednrb*^*fl/fl*^, and Cag-creER^T2+^*Ednra*^*fl/fl*^ animals. Full body deletion of *Ednra* ablated EDN1-induced vasoconstriction (*n* ≥ 5).

**Fig. 3:**
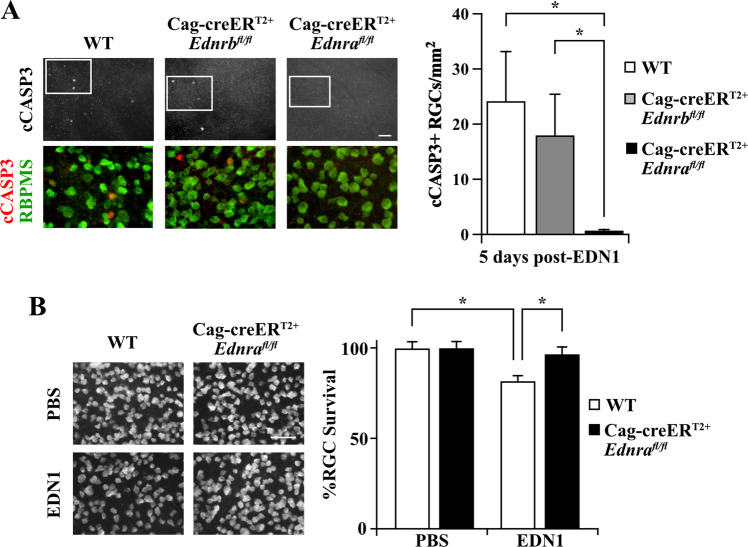
EDN1 ligand acted through EDNRA to drive RGC death. **A** Retinal flat mounts from WT, Cag-creER^T2+^*Ednrb*^*fl/fl*^, and Cag-creER^T2+^*Ednra*^*fl/fl*^ mice immunoassayed for cCASP3 and RBPMS 5 days post-EDN1. Cag-creER^T2+^*Ednrb*^*fl/fl*^ retinas had similar numbers of cCASP3+ RGCs compared to WT controls, while Cag-creER^T2+^*Ednra*^*fl/fl*^ mice had significantly reduced cCASP3+ RGCs compared to both WT and Cag-creER^T2+^*Ednrb*^*fl/fl*^ retinas. cCASP3+ RGCs/mm^2^ ± SEM: WT: 24.2 ± 8.9 Cag-creER^T2+^*Ednrb*^*fl/fl*^: 18.0 ± 7.4, Cag-creER^T2+^*Ednra*^*fl/fl*^: 0.7 ± 0.1 (*n* ≥ 6, *P < 0.01, Kruskal-Wallis test). Scale bar, 50 μm. **B** Flat mounted WT and Cag-creER^T2+^*Ednra*^*fl/fl*^ retinas immunoassayed for RBPMS 28 days post-EDN1 injection. Full body deletion of *Ednra* prevented EDN1-induced RGC loss. %RBPMS+ cell survival±SEM for WT and Cag-creER^T2+^*Ednra*^*fl/fl*^ respectively: PBS: 100.0 ± 3.4, 100.0 ± 2.7; EDN1: 82.0 ± 2.7, 97.0 ± 3.8 (*n* ≥ 6, *P < 0.05, two-way ANOVA, Holm–Sidak post hoc). Scale bars, 50 μm.

Cag-creER^T2+^TdTomato^+^ retinas and optic nerves were first evaluated to assess Cag-creER^T2^ recombination efficiency. Cag-creER^T2+^TdTomato^+^ retinas had TdTomato expression in nearly all DAPI+ cells in the retina, including retinal neurons, macroglia, and vascular cells (Fig. 2A). Furthermore, EDN ligand is known to induce vasoconstriction upon binding to vascular mural cell-expressed EDNRA [8–11]. Cag-creER^T2^-mediated deletion of *Ednra* completely prevented retinal vasoconstriction in response to intravitreal EDN1 (Fig. 2B). Therefore, EDN receptors were successfully deleted from the major cell types in the retina and optic nerve known to endogenously express each receptor. To determine whether EDN ligand binds to EDNRA or EDNRB to ultimately elicit RGC death, EDN1 was intravitreally injected into both Cag-creER^T2+^*Ednra*^*fl/fl*^ and Cag-creER^T2+^*Ednrb*^*fl/fl*^ mice. Global deletion of *Ednra*, but not *Ednrb*, prevented EDN1-induced caspase 3 activation in RGCs 5 days following injury (Fig. 3A). Furthermore, global *Ednra* deletion prevented RGC loss 28 days post-EDN1 (Fig. 3B). These data suggest, in contrast to previous reports [12, 25], EDN ligands act through non-neuronal/macroglial cell types expressing EDNRA to ultimately cause RGC death.

### Endothelin ligand caused RGC death via mural cell-expressed EDNRA

**Fig. 4:**
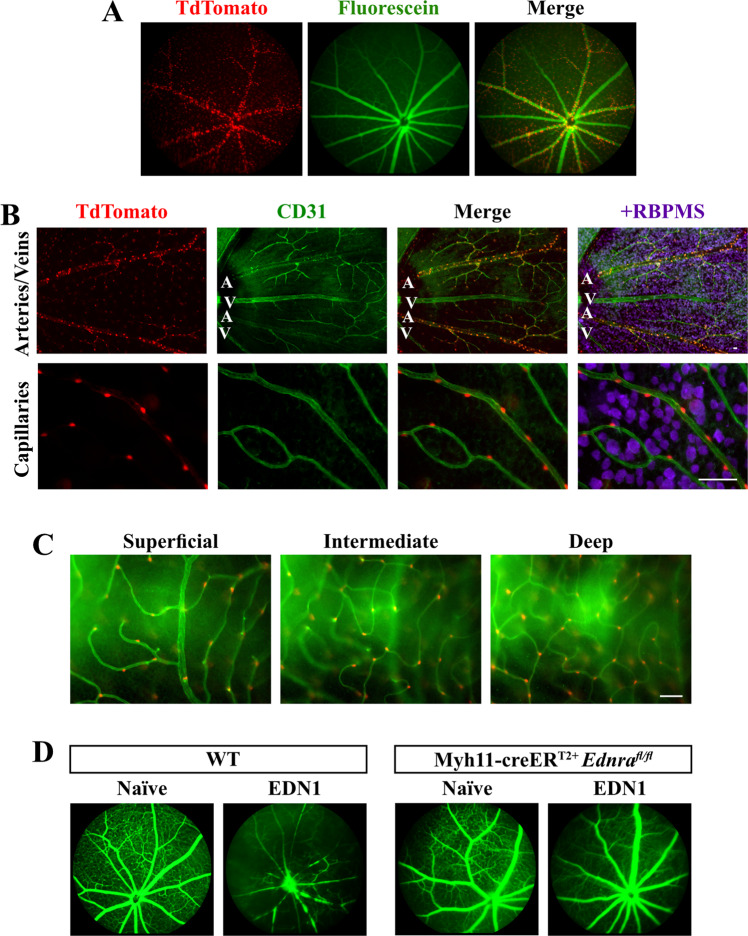
Myh11-creER^T2^ recombined floxed alleles in vascular mural cells. **A** Myh11-creER^T2+^Tdtomato^+^ fluorescein angiography overlayed with TdTomato fluorescence demonstrating TdTomato localization to retinal arteries (*n* = 4). **B** Myh11-creER^T2^Tdtomato^+^ retinal flat mounts counterstained with CD31 to visualize retinal vasculature. Tdtomato was robustly and specifically expressed by arterial cells and capillaries, but not by RBPMS+ RGCs or any other observable cell type. **C** Tdtomato+ cells surrounding retinal capillaries were apparent in superficial, intermediate, and deep layers of the retina (*n* = 3). Scale bars, 50 μm. **D** Fluorescein angiography of retinal vasculature in naive and EDN1-injected eyes from WT and Myh11-creER^T2+^*Ednra*^*fl/fl*^ animals. Mural cell-specific deletion of *Ednra* ablated EDN1-induced vasoconstriction (*n* ≥ 5).

**Fig. 5:**
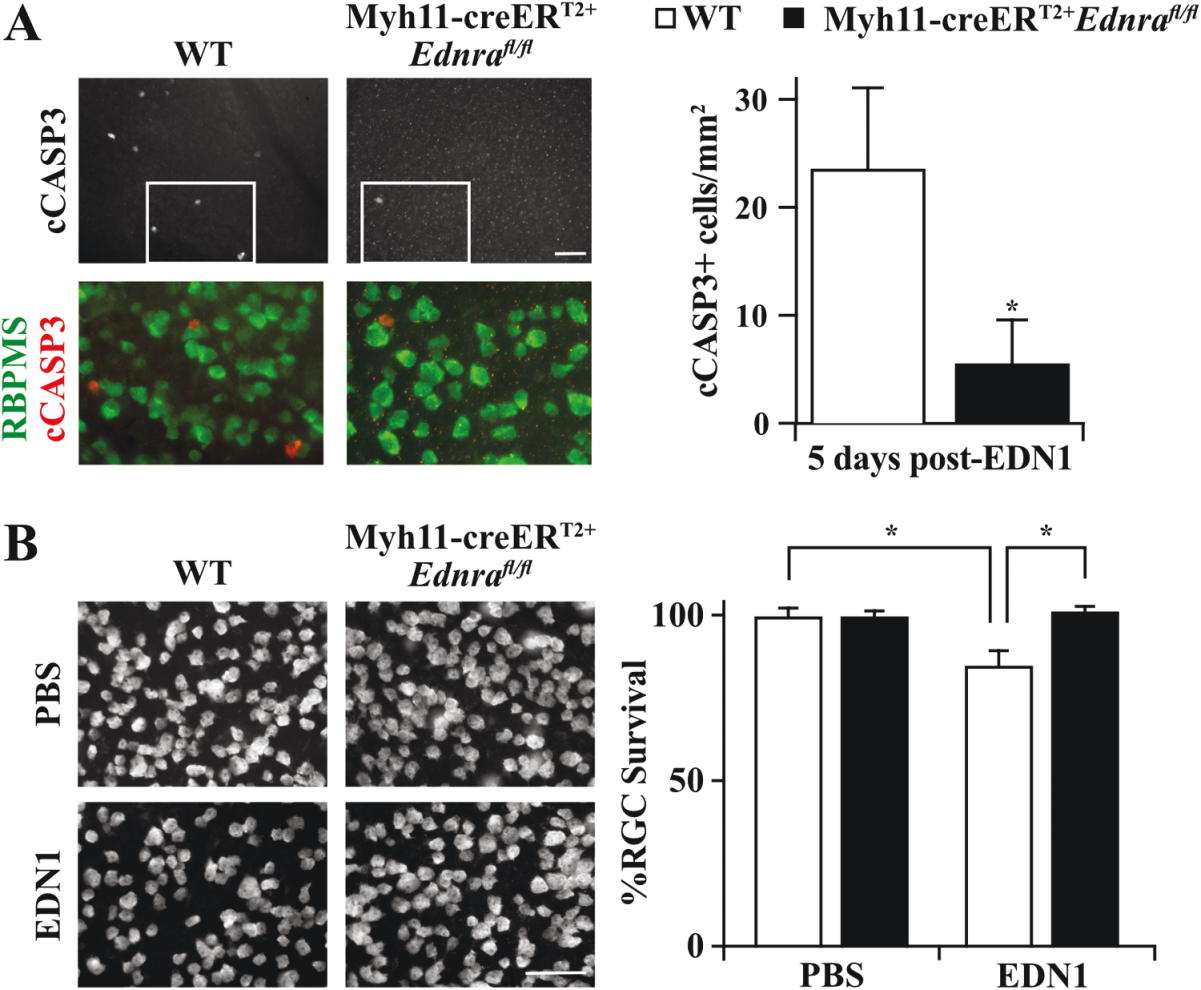
EDNRA expressed by vascular mural cells elicited RGC death in response to EDN1. **A** Flat mounted retinas immunoassayed for cCASP3 and RBPMS 5 days post-EDN1 injection. *Ednra* deletion from vascular mural cells significantly reduced numbers of cCASP3+ RGCs after EDN1 injury. cCASP3+ RGCs/mm^2^: WT: 23.6 ± 7.6, Myh11-creER^T2+^*Ednra*^*fl/fl*^: 6.0 ± 4.4 (*n* ≥ 9, *P = 0.009, Mann–Whitney test). **B** Flat mounted WT and Myh11-creER^T2+^*Ednra*^*fl/fl*^ retinas immunoassayed for RBPMS 28 days post-EDN1 injection. Mural cell deletion of *Ednra* prevented EDN-induced RGC loss. %RBPMS+ cell survival ± SEM for WT and Myh11-creER^T2+^*Ednra*^*fl/fl*^ respectively: PBS: 100.0 ± 2.3, 100.0 ± 1.4; EDN1: 85.0 ± 4.3, 101.4 ± 1.3 (*n* ≥ 8, *P ≤ 0.001, two-way ANOVA, Holm–Sidak post hoc). Scale bars, 50 μm.

Full-body deletion of *Ednra* prevented both EDN1-induced vasoconstriction and RGC death. Beyond expression by RGCs and astrocytes, EDNRA is expressed by vascular mural cells. Upon ligand binding, vascular mural cell EDNRA elicits contraction (vasoconstriction) [8–11]. Therefore, the role of vascular mural cell EDNRA in RGC death in response to EDN ligand was investigated. To accomplish this, *Ednra*^*fl*^ alleles were recombined from vascular mural cells (vascular smooth muscle cells and pericytes) with Myh11-creER^T2^ upon tamoxifen treatment [51, 52]. Myh11-creER^T2+^TdTomato^+^ retinas were first evaluated to assess cre recombination efficiency and specificity in retinal vascular cells (Fig. 4A–C). In vivo angiography and ex vivo immunofluorescence revealed TdTomato expression was specifically localized to retinal arteries (identified by distinct branching pattern compared to retinal veins [53]) and capillaries in superficial, intermediate, and deep layers of the retina. Importantly, EDN1-induced vasoconstriction was attenuated in Myh11-creER^T2+^*Ednra*^*fl/fl*^ retinas (Fig. 4D). Therefore, *Ednra*^*fl*^ alleles were specifically and efficiently recombined from retinal vascular mural cells. To determine whether EDN acts through vascular mural cell-expressed EDNRA to cause RGC death, EDN1 ligand was intravitreally injected into Myh11-creER^T2+^*Ednra*^*fl/fl*^ mice. Mural cell deletion of *Ednra* prevented caspase 3 activation in RGCs 5 days after EDN1 (Fig. 5A), and prevented RGC loss after 28 days (Fig. 5B). Therefore, EDN1 ligand acted through EDNRA expressed by mural cells to ultimately drive RGC death.

## Discussion

Glaucoma is a multifactorial, heterogeneous neurodegenerative condition. Often In glaucoma, an increase in IOP leads to RGC injury and subsequent death. Several hypotheses have been postulated as to how ocular hypertension leads to RGC injury in glaucoma. Recent work has provided strong evidence for the role of endothelin signaling in causing RGC injury and subsequent death in models of chronic ocular hypertension [5, 6]. EDN ligands were upregulated in human [34, 54] and animal models [3, 5, 25, 40] of glaucoma, and intravitreal injection of EDN ligand was sufficient to drive caspase 3 activation in RGCs, which corresponded with later RGC death [44]. Similar to models of glaucoma-relevant axonal injury [45] and ocular hypertension [46], deletion of *Jun* from RGCs prevented caspase 3 activation in RGCs and prevented later RGC loss after intravitreal EDN1 injection [44]. Pan-antagonism of EDN receptors significantly slowed RGC loss in the DBA/2J model of ocular hypertension [5, 6], suggesting a causal role for the endothelin system in glaucoma pathogenesis. However, it was unknown which cell type EDN ligands directly affect in order to ultimately drive RGC death, and through which receptor (EDNRA or EDNRB) this occurs.

Previous in vitro studies have suggested EDN ligands can cause primary RGC death, suggesting EDN ligands are directly neurotoxic to RGCs (acting through RGC-expressed EDN receptors) [12]. Here, we demonstrate EDN1 ligands did not cause RGC death directly and did not cause RGC death by affecting other retinal neurons or macroglia in vivo (Fig. 1). Also, in contrast with previous studies suggesting EDN ligands act through EDNRB to drive RGC death in vitro, after EDN injection in vivo, and in a model of chronic ocular hypertension [12, 25], EDNRB was not required for EDN1-induced RGC death. Rather, EDNRA was the receptor that was necessary for EDN1-induced RGC death (Fig. 3). Given the canonical role of EDNRA is to mediate vasoconstriction [8–11], we investigated the importance of mural cell-expressed EDNRA in EDN-induced RGC death. We demonstrated EDN1-induced RGC death was driven by vascular mural cell (smooth muscle and pericyte)-expressed EDNRA (Fig. 5). These data do not preclude the possibility that vascular mural cells respond to EDN1 by eliciting neurotoxic paracrine or endocrine signaling. However, it is likely EDN1-induced RGC death is a result of EDNRA-mediated vasoconstriction and its sequalae.

Because *Edn* ligands were upregulated in DBA/2J glaucoma [3, 5, 7], pan-antagonism of EDN signaling lessened RGC loss after ocular hypertensive insults [5, 6], and EDN1-induced RGC death was driven by vascular mural cell-expressed EDNRA (Fig. 5), it is possible that chronic vascular pathology or vasoconstriction is an important mediator of RGC death in glaucoma. Vascular involvement is consistent with several observations in human and animal models of glaucoma. Reduced ocular and retinal blood flow have been documented in human [36–39] and animal models [3, 5] of glaucoma, and it is hypothesized that these changes could be important factors in the development and progression of glaucoma. Hypoxic glia and RGCs were present after acute [55, 56] and chronic [57] ocular hypertension in rodents, suggesting the potential importance of hypoxia in driving glaucoma-relevant pathology.

If vascular EDNRA-induced vasoconstriction causes RGC death in response to EDN ligand, it will be important to investigate the pathological cellular events that lead from vasoconstriction to RGC death. While EDN1 injection was shown to cause regional RGC and glial hypoxia [44] (similar to glaucomatous ocular hypertension [55, 57]), oxygen deprivation itself is unlikely to cause this RGC death. Oxygen deprivation severe enough to cause RGC death is also known to cause loss of amacrine neurons [58–62]. Previous work has demonstrated that, similar to ocular hypertension [63], EDN1 injection was not sufficient to drive the death of amacrine cells [44]. Therefore, if vasoconstriction is important in EDN-induced RGC death, it most likely drives secondary neurotoxic pathological events.

Chronic low-level hypoxia mediated by endothelin signaling may lead to compromise of the blood-brain barrier after EDN1 exposure and in glaucoma. In vitro, hypoxic conditions were sufficient to degrade endothelial cell tight junctions and cause barrier permeability [64–66]. Chronic mild hypoxia [67] and transgenic overexpression or injection of EDN ligand [43, 68, 69] led to loss of blood–brain barrier integrity and vascular leakage in vivo. Breakdown of the blood-brain barrier and subsequent infiltration of peripheral immune cells has been suggested to drive neurodegeneration in glaucoma—prevention of immune cell infiltration with radiation therapy protected from glaucoma in DBA/2J mice [3]. Similarly, deletion of *Cd11b* (also known as *Itgam*—a cell adhesion protein critical for tissue infiltration of monocytes) was shown to lessen monocyte infiltration into the optic nerve head and protect from glaucomatous neurodegeneration in DBA/2J mice [70]. Consistent with these results, deletion of *Glycam* (a proteoglycan ligand for L-selectin known to prevent transendothelial migration of leukocytes) promoted monocyte infiltration into the optic nerve head and weakened the protection afforded to DBA/2J retinas by radiation treatment [4]. Given the importance of vascular compromise in glaucoma, it is possible that EDNRA-induced vasoconstriction and its sequelae damages the blood-brain barrier and plays a role in neurodegeneration in response to EDN1 ligand and in glaucoma.

It is also possible that EDN-induced regional mild hypoxia can affect immune cells in the retina or optic nerve. Astrocytes took on a reactive phenotype in response to hypoxia in vitro [71, 72] and in vivo [73, 74], which could potentially lead to a neurotoxic gliotic response. Furthermore, astrocytes aid in maintaining blood-brain barrier integrity. Chronic hypoxic conditions led to a loss of astrocyte-endothelial cell contacts [65]. Astrocytes are also known to upregulate and secrete VEGF upon hypoxic insult [75]. Astrocyte-specific VEGF was critical for pathological neovascularization after retinal hypoxic injury in vivo [76]. VEGF was required for hypoxia-induced blood-brain barrier breakdown [66], and astrocyte-specific VEGF was shown to cause blood–brain barrier breakdown in vitro [77]. Together, these data suggest hypoxia can cause changes in retinal astrocytes, which can in turn drive neurotoxic signaling and/or contribute to breakdown of the blood–brain barrier. The mechanisms by which EDN-EDNRA signaling drive RGC death must be elucidated, and the importance of these events in driving glaucomatous neurodegeneration upon chronic ocular hypertension merits future investigation.

## Materials and methods

### Mice

All mice used were 1.5–6 months of age. Mice were fed chow and water ad libitum and housed on a 12-hour light-to-dark cycle. All experiments were conducted in adherence to the Association for Research in Vision and Ophthalmology’s statement on the use of animals in ophthalmic and vision research and were approved by the University of Rochester’s University Committee on Animal Resources. C57BL/6N-At^*m1Brd*^*Ednra*^*tm1a(EUCOMM)Hmgu*^/JMmucd knockout-first mice with promoter-driven alleles were obtained through UC Davis KOMP Repository. These mice were crossed with flippase recombinase transgenic mice (Flp^tg^, URMC genomics research core) to generate offspring with *Ednra*^*fl*^ alleles. *Ednrb*^*tm1.1Nat*^/J alleles were obtained from the Jackson Laboratory (*Ednrb*^*fl*^; Stock #011080 [47]). Mice with *Ednra*^*fl*^ and *Ednrb*^*fl*^ alleles were bred to Tg(Six3-cre)69Frty/GcoJ transgenic mice (Six3-cre^+^; Jackson Laboratory, Stock# 019755) [48] to generate mice with a conditional deletion of *Ednra* and/or *Ednrb* from retinal neurons and macroglia. Mice with *Ednra*^*fl*^ or *Ednrb*^*fl*^ alleles were also bred to Tg(CAG-cre/Esr1*)5Amc/J transgenic mice (Cag-creER^T2+^; JAX Stock #004682) [78] to produce offspring with full-body deletions of *Ednra* or *Ednrb* upon tamoxifen treatment. Mice with *Ednra*^*fl*^ alleles were bred to Tg(Myh11-cre/ERT2)1Soff/J transgenic mice (Myh11-creER^T2+^; Jackson Laboratory, Stock# 019079) [79] to generate mice with a conditional deletion of *Ednra* from vascular mural cells upon tamoxifen treatment. Mice transgenic for Cag-creER^T2^ or Myh11-creER^T2^ recombinase were bred to *Gt(ROSA)26Sor*^tm75.1(CAG-tdTomato*)Hze^/J conditional reporter mice (TdTomato^+^; JAX stock 025106) to generate offspring as TdTomato reporters of cre expression.

### Statistical analysis and experimental rigor

Power calculations were performed before experiments were conducted to determine the appropriate sample size. Data were analyzed using GraphPad Prism9 software. Data from experiments designed to test differences between two groups were subjected to an F test to compare variance and a Shapiro-Wilk test to test normality to ensure appropriate statistical tests were utilized. For non-normally distributed data designed to test differences between two groups, a Mann–Whitney test was utilized. Data from experiments designed to test differences among more than two groups across one condition were subjected to a Brown–Forsythe test to compare variance and a Shapiro–Wilk test to test normality to ensure an appropriate statistical test was utilized. Data from experiments designed to detect differences among multiple groups and across one condition were analyzed using a Kruskal–Wallis test with Dunn’s post hoc test. Data from experiments designed to detect differences among multiple groups and across two conditions were analyzed using a two-way analysis of variance followed by Holm–Sidak’s post hoc test. For these statistical tests, every possible comparison was made when relevant, and multiplicity adjusted *P* values are reported. In all cases, data met the assumptions of the statistical test used. *P* values < 0.05 were considered statistically significant. Throughout the manuscript, results are reported as mean ± standard error of the mean (SEM).

Roughly equal numbers of male and female mice were used for each experimental group, except for Myh11-creER^T2^*Ednra*^*fl*^ mice (the Myh11-creER^T2^ transgene is inserted into the Y chromosome, thus, all animals used for this line of experiments were male). Phenotypically wild-type (WT) controls included tamoxifen treated and untreated cre^+^ and cre^−^ animals. Littermate controls were used wherever possible. Animals were randomly assigned to experimental groups. Before experiments were performed, it was established that animals with pre-existing abnormal eye phenotypes (e.g., displaced pupil, cataracts) would be excluded from the study. All procedures were conducted by an observer masked to genotype and condition.

### Tamoxifen treatment and animal procedures

At 6 weeks of age or older, animals were intraperitoneally injected with 125 mg/kg tamoxifen (Sigma, T5648) dissolved in corn oil at a concentration of 20 mg/mL once per day for five consecutive days. Experiments were conducted no earlier than 7 days after the last tamoxifen dose to allow for recombination of floxed alleles and degeneration of endogenous protein. Intravitreal injections and fluorescein angiography were performed as previously described [44]. EDN1 (Sigma, E7764) was dissolved in sterile PBS at a concentration of 500 μM. As previously performed, 2 μL of 500 μM EDN1 dissolved in sterile PBS was intravitreally injected into one eye. Sterile PBS was injected into the contralateral eye as a volume-matched vehicle control.

### Tissue processing, immunofluorescence, and cell quantification

Tissue processing, immunostaining, and cell quantification were performed as previously described [44] using the following primary antibodies: rabbit anti-cCASP3 (R&D Systems, AF835, 1:1000), rabbit anti-RBPMS (GeneTex, GTX118619, 1:250), guinea pig anti-RBPMS (PhosphoSolutions, 1832-RBPMS, 1:250), goat anti-SOX2 (Santa Cruz, sc-17320, 1:200), goat anti-CD31 (R&D Systems, AF3628, 1:1000), and chicken anti-GFAP (Abcam, ab4674, 1:500).

## Supplementary information

Author Contribution form

## Data Availability

The datasets used in the current study are available from the corresponding author on reasonable request.
