# Supplementary material for: Vascular derived endothelin receptor A controls endothelin-induced retinal ganglion cell death
Source: Cell Death Discov. 2022 Apr 16;8:207. doi: 10.1038/s41420-022-00985-8 (PMC9013356; doi:10.1038/s41420-022-00985-8)
Supplement: Supplementary file 1 — Author Contribution form [file 41420_2022_985_MOESM1_ESM.pdf]

DECLARATION OF CONTRIBUTIONS TO ARTICLE

ADMC

Manuscript Number: CDDISCOVERY-22-3685

Journal Name: *Cell Death Discovery* (the 'Journal')

Proposed Title of the Contribution:

Vascular derived endothelin receptor A controls endothelin-induced retinal ganglion cell death (the 'Contribution)

Author(s):

Olivia J. Marola, Gareth R. Howell, and Richard T. Libby (the 'Authors')

For all *CDDis*covery articles, each person named as an author in the published version must be able to show he or she has contributed substantially to the article.

Authorship credit should be based on 1) substantial contributions to conception and design, acquisition of data, or analysis and interpretation of data; 2) drafting the article or revising it critically for important intellectual content; and 3) final approval of the version to be published. Authors should meet conditions 1, 2 and 3.

Any person who cannot be shown to have made a substantial contribution to the article cannot be listed as an author in the final version. The name of any person who is deemed to have made a minor contribution can, however, appear in the Acknowledgments section of the article.

Please complete the table below to indicate the contributions of all named authors to the manuscript.

Author Full Name:	Specification of Contribution to the Manuscript:
Olivia J. Marola	Designed experiments, acquired data, analyzed and interpreted data, drafted the manuscript
Gareth R. Howell	Conceived of and designed experiments, aided in drafting and revising the manuscript
Richard T. Libby	Conceived of and designed experiments, aided in drafting and revising the manuscript

Please complete the table below to indicate the contributions of all named authors to the figures.

Figure 1:
OJM acquired and analyzed data.

Figure 2:
OJM acquired and analyzed data

Figure 3:
OJM acquired and analyzed data

Figure 4:
OJM acquired and analyzed data

Figure 5:
OJM acquired and analyzed data

Figure 6:

Signed for and on behalf of the Author(s):	Print Name:	Date:
	Richard T Libby	2/1/22
